# Integrated Fiber Ring Laser Temperature Sensor Based on Vernier Effect with Lyot–Sagnac Interferometer

**DOI:** 10.3390/s23146632

**Published:** 2023-07-24

**Authors:** Yuhui Liu, Weihao Lin, Jie Hu, Fang Zhao, Feihong Yu, Shuaiqi Liu, Jinna Chen, Huanhuan Liu, Perry Ping Shum, Xuming Zhang

**Affiliations:** 1 Department of Electrical and Electronic Engineering, Southern University of Science and Technology, Shenzhen 518055, China; 12068026@mail.sustech.edu.cn (Y.L.); 11510630@mail.sustech.edu.cn (W.L.); 12031313@mail.sustech.edu.cn (J.H.); 12031197@mail.sustech.edu.cn (F.Z.); 11930480@mail.sustech.edu.cn (F.Y.); 11853004@mail.sustech.edu.cn (S.L.); chenjn@sustech.edu.cn (J.C.); liuhh@sustech.edu.cn (H.L.); shenp@sustech.edu.cn (P.P.S.); 2 Department of Applied Physics, Hong Kong Polytechnic University, Hongkong 999077, China; 3 Peng Cheng Laboratory, Shenzhen 518005, China

**Keywords:** temperature sensor, Lyot–Sagnac loop, fiber ring laser

## Abstract

The Vernier effect created using an incorporated Lyot–Sagnac loop is used to create an ultra-high sensitivity temperature sensor based on a ring laser cavity. Unlike standard double Sagnac loop systems, the proposed sensor is fused into a single Sagnac loop by adjusting the welding angle between two polarization-maintaining fibers (PMFs) to achieve effective temperature sensitivity amplification. The PMFs are separated into two arms of 0.8 m and 1 m in length, with a 45° angle difference between the fast axes. The sensor’s performance is examined both theoretically and experimentally. The experimental results reveal that the Vernier amplification effect can be achieved via PMF rotating shaft welding. The temperature sensitivity in the laser cavity can reach 2.391 nm/°C, which is increased by a factor of more than eight times compared with a single Sagnac loop structure (0.298 nm/°C) with a length of 0.8 m without the Vernier effect at temperatures ranging from 20 °C to 30 °C. Furthermore, unlike traditional optical fiber sensing that uses a broadband light source (BBS) for detection, which causes issues such as low signal-to-noise ratio and broad bandwidth, the Sagnac loop can be employed as a filter by inserting itself into the fiber ring laser (FRL) cavity. When the external parameters change, the laser is offset by the interference general modulation, allowing the external temperature to be monitored. The superior performance of signal-to-noise ratios of up to 50 dB and bandwidths of less than 0.2 nm is achieved. The proposed sensor has a simple structure and high sensitivity and is expected to play a role in biological cell activity monitoring.

## 1. Introduction

Fiber optic temperature sensors have received a lot of interest in recent years due to their benefits, including their small size, lightweight, resilience to corrosion, and immunity to electromagnetic interference [1,2,3,4,5]. Various interference structures, including the Mach–Zehnder interferometer (MZI) [6,7,8], Fabry–Perot interferometer (FPI) [9,10] and Sagnac loop [11,12], are designed to monitor temperature. MZI refers to a type of structure in which light is divided into two beams passing through different paths separately, resulting in a relative phase shift followed by interference at the convergence point. The incident light and the outgoing light pass through the different interfaces of the MZI and do not interact with each other. Using the core offset style of an MZI, a temperature sensitivity of 0.0462 nm/°C was achieved [13]. Lin et al. used double peanuts structure as an MZI and realized a sensitivity of temperature measurement of 1.038 nm/°C [1]. If the incident light and the outgoing light pass through the same way, it is called FPI. Typically, it contains elements with high emissivity in the structure where incident light is reflected. Then the reflection light interferes with incident light, and the interference result is subsequently detected. For example, Wu et al. etched side hole fiber to form an open-cavity FPI and successfully tested the temperature change with a sensitivity of −6.14 pm/°C [14]. As for the Sagnac loop, two coherent lights transmitted in clockwise and counterclockwise directions are superimposed to create interference. A Sagnac loop composed of photonic crystal fiber with selective ethanol-filled sections was designed for temperature sensing, and the temperature sensitivity was 1.65 nm/°C ranging from 25 to 33 °C [15]. Among them, detection sensitivity is always a crucial technical issue to be resolved and discussed because of the limitations of optical fiber qualities.

The optical Vernier effect, which amplifies minute optical offsets, is frequently exploited in optical fiber sensing to address this issue [16,17,18,19]. Upon combining a micro-nano fiber coupler with magnetic fluid, Yuan et al. achieved Vernier amplification [20], increasing the magnetic field sensitivity to −98 nm/mT. Yet, because they trim the fiber to the micron scale, micro-nano fiber couplers have poor stability and repeatability. In order to create an FPI structure for airflow monitoring, Zhao et al. combined coreless fiber and single-mode fiber [21], which increased the sensing sensitivity by 9.57 times. However, the system possesses a wide bandwidth and a low signal-to-noise ratio. For the dual parameter measurement of pressure and temperature, Andre D. Gomes et al. created a hollow spherical FPI structure [22], attaining a temperature response of −650 pm/°C. The method is difficult, unrepeatable, and insufficiently sensitive. Recently, the sensitivity of temperature sensors has been improved to the order of ~nm/°C or even tens of nm/°C through more complex structures [23,24,25,26]. However, these experiments based on the Vernier effect require at least two parts of the sensing structure, which means poor repeatability due to high structural and process complexity. At the same time, the detection range, as well as the signal-to-noise ratio, are limited, resulting in suboptimal results in practical applications.

Fiber optic Sagnac loop interferometers have been widely studied and used since they were proposed [27,28,29]. Served as a fiber optic gyroscope, the Sagnac loop displays good performance in simple structures, high reliability, wide dynamic range, and low cost. Another important application of the Sagnac loop is fiber optic filters. Upon adding different fiber structures to the Sagnac loop, such as FBGs, PMFs, or fiber optic ring resonators, the role of filtering can be achieved. In general, the addition of a mere section of PMF to attain the resulting structure is not enough to provide sufficient sensitivity. Hence, the Lyot–Sagnac interferometer was designed to achieve the Vernier effect for sensing. The Lyot–Sagnac interferometer comprises multi-period high birefringence fibers and a polarization controller connected within a single Sagnac loop. The high-order high birefringence fiber Sagnac interferometer is commonly abbreviated as the n-order Lyot–Sagnac interferometer [30,31].

In the past ten years, fiber optic sensors based on fiber ring laser (FRL) systems have been greatly developed [32,33,34]. Compared with a broadband light source (BBS), it has a higher signal-to-noise ratio, better bandwidth, and better detection resolution. Liu et al. proposed to use of photonic crystal fiber to fill liquid crystal material and indium tin oxide (ITO) conductive glass to detect electric fields in an FRL system. A detection sensitivity of 1.1 nm/Vrms was achieved, and the signal-to-noise ratio was as high as 35 dB [35]. Hu et al. designed a Michelson interferometric microfluidic channel for chemical detection [36], which increased the detection limit to 0.06 mg/mL, and the laser bandwidth was less than 0.09 nm. In addition, our research group proposed to use double Saganc loops for the first time to realize the Vernier amplification effect in an FRL system. The temperature sensitivity is improved to the order of 4 nm/°C [37].

In this study, we propose a novel FRL temperature sensing system based on a Lyot–Sagnac loop to achieve Vernier amplification. Unlike cascading Sagnac loops where double loops are required, this proposed structure saves half of the material compared with double loops. To simplify the structure, PMFs with lengths of 0.8 m and 1 m were inserted in a single Sagnac loop, and the two ends of PMFs were fused by rotating axes, forming a 45° angle between axes to accomplish the vernier magnifying effect. The experimental results reveal that the Lyot structure has a better temperature response (2.391 nm/°C) than the single loop (−0.298 nm/°C), in which the sensitivity is effectively increased by eight times. At the same time, because of the benefits of the FRL system, the developed temperature sensing system has a signal-to-noise ratio greater than 50 dB and a bandwidth smaller than 0.2 nm. The proposed sensor has the advantages of a simple structure, low cost, and good stability. It has great potential for temperature monitoring in the range of small temperature changes.

In the following part of this study, we will show the experimental setup, explain the working principle of the designed structure, demonstrate the experimental process, present the experimental results, and analyze them in turn. At the end of the study is the summary of the whole study.

## 2. Experimental Setup and Working Principle

**Figure 1:**
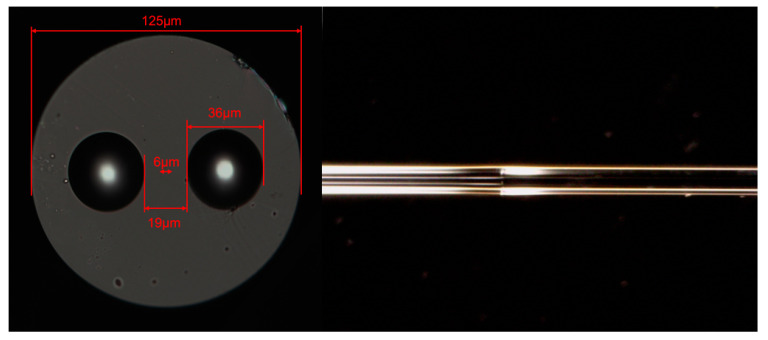
Cross section diagram of polarization maintaining fiber is shown on the left, and the fiber splice joint diagram is on the right.

In this experiment, panda-type PMF (PM # 1550_125-18/250, YOFC, Shanghai, China) is utilized, and the cross-sectional diagram is shown left side in Figure 1. The total diameter of this PMF is 125 µm with a measured fiber core of about 6 µm. The two stress zones, symmetrical with the fiber core, are 19 µm apart and have a diameter of 36 µm. At first, two sections of panda PMF with a length of 0.8 m and 1 m were intercepted. Later, the two PMFs are spliced by polarization-maintaining fiber splicer (FSM-100P+, Fujikura, Tokyo, Japan) with an angle difference of 45° between the fast axes. The detailed operation procedure is flattening the end faces of the two PMFs using a cutter and placing them in two rotating fixtures of the polarization-maintaining fiber splicer. After cleaning using discharge, a microscope is tuned to focus on the fiber end face, where the PMF cross-sectional image can be clearly seen. Then adjust and secure the two sections of fiber to form a 45° rotation angle. After discharge welding, the target fiber can be obtained, as shown on the right side of Figure 1.

**Figure 2:**
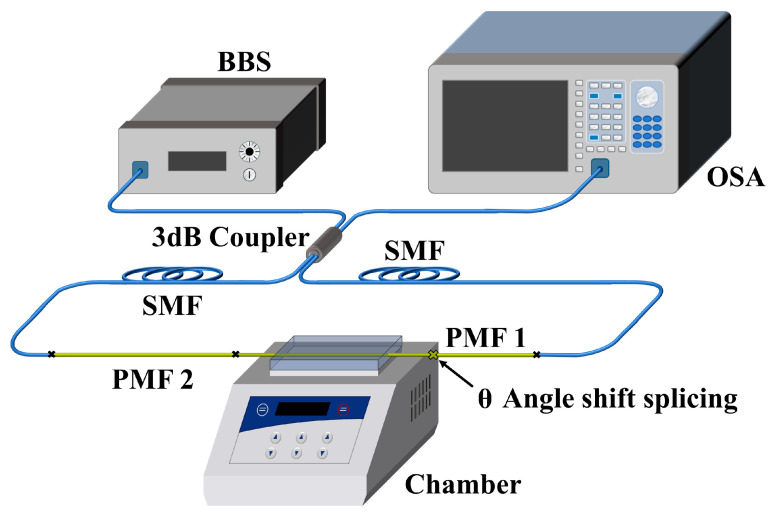
Schematic diagram of the experimental setup for the temperature detection system in BBS.

In order to verify that the Lyot–Sagnac loop can achieve the Vernier amplification effect, a pre-experiment is needed. The pre-experimental device is shown in Figure 2. Part of the 1 m length PMF is attached securely to the temperature control platform of a commercial temperature controller (MK-20, Allsheng, Hangzhou, China) with high-temperature tape. The temperature of this commercial temperature controller has been unified calibration to an accuracy of 0.1 °C. The temperature-controlled zone is made of metal, making the temperature of this area reach the target temperature quickly and uniformly after setting different temperature parameters. The BBS (Hoyatek ASE-C-N) with a power of 10 mW enters the PMFs with a length of 0.8 m and 1 m at the same time through the 3 dB coupler forward and reverse, the rotation angle between the two PMFs is 45° and then returns to the coupler through the loop to input into the optical Spectrum Analyzer (OSA, Yokogawa AQ6370D, Yokogawa, Tokyo, Japan, bandwidth: 600 nm–1700 nm; resolution: 0.01 nm) to read interference data.

**Figure 3:**
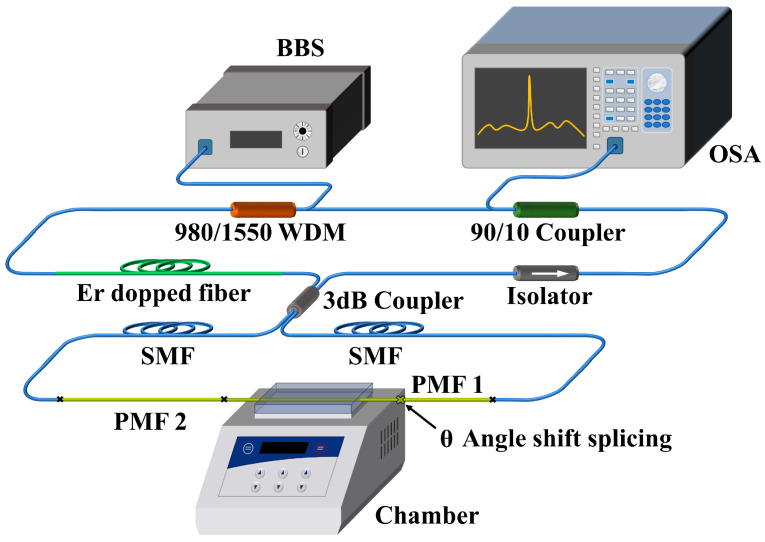
Schematic diagram of the experimental setup for the temperature detection system in the FRL system.

Next, we put the Lyot–Sagnac loop into the FRL cavity. Its schematic diagram is shown in Figure 3. The 980 nm pump source generates laser through the gain medium (erbium-doped fiber) pumped annular cavity, which is modulated using the Sagnac loop and shifts the laser output peak under external temperature change. The isolator is used to prevent backscattered light from damaging the device. After the 90/10 coupler, most of the light energy is continuously circulated in the cavity, and 10% of the energy is input into the OSA internal for reading data.

The output light is fed into the 3 dB coupler and transmitted in both clockwise and counterclockwise directions. The propagation distance difference is due to the fact that the PMF has the refractive index difference generated by the fast and slow axes. The input light will produce a phase difference after passing through the first part of PMF. At the same time, because the axial spacing between the two PMFs is about 45° when the light passes through the second part of the PMF, it will produce an additional phase difference again. Therefore, the output projected spectrum is produced by superimposing two interference spectra with approximately periodic periods, which are affected by the Vernier effect. The expression of its output spectrum is [38]:

(1) $$T={\left[\cos\left(\varphi_{1}+\varphi_{2}\right)\cos\theta +\cos\left(\varphi_{1}-\varphi_{2}\right)\sin\theta \right]}^{2}$$

In which, φ1=2πBL1λ, φ2=2πBL2λ represent the different phase condition in PMF 1 and PMF 2. The birefringence of PMFs is expressed by B. λ is the output light’s wavelength. $L_{1}$ and $L_{2}$ means the lengths of PMF 1 and PMF 2. The included angle between the fast axes of the two PMFS is θ. Because of the optical Vernier effect, the period S of the envelope can be expressed as [38]:

(2) $$S=\frac{{FSR}_{1}\times {FSR}_{2}}{\left|{FSR}_{1}-{FSR}_{2}\right|}=\frac{\lambda }{B\left|L_{2}-L_{1}\right|}$$

Wherein the free spectral range (FSR) of two spectra with different periods is represented by ${FSR}_{1}$ and ${FSR}_{2.}$

(3) $${FSR}_{1}=\frac{\lambda^{2}}{BL_{1}},{FSR}_{2}=\frac{\lambda^{2}}{BL_{2}}$$

Two PMFs serve as measuring calipers, the main caliper and the Vernier caliper. The maximum interference peak output occurs when the scales between the two scales are flush. If the interference peak on PMF 1 is offset by temperature modulation, the entire Lyot–Sagnac loop will be amplified. Therefore, the overall envelope offset can be represented by the amplification factor A [38]:

(4) $$A=L_{1}/\left|L_{2}-L_{1}\right|$$

The resonant wavelength $\lambda_{dip}$ of heating PMF 1 can be expressed as if the heating range is $l_{1}$ [34]:

(5) $$\lambda_{dip}=\frac{BL_{1}+\Delta Bl_{1}+B\Delta l}{k}$$

The dip wavelength shift $\Delta \lambda_{dip}$ can be described as [34]:

(6) $$\Delta \lambda_{dip}=\frac{\Delta Bl_{1}+B\Delta l}{k}$$

The change in birefringence caused by the change in external temperature is ΔB=aΔT, a is the thermo-optical coefficient of the fiber. The change in fiber length caused by temperature change is Δl=βΔT, β is the coefficient of thermal expansion. Hence, the wavelength shift caused by temperature change can be expressed as [38]:

(7) Δλdip=aB+βl1·λdip·ΔT

In view of the above expression, the temperature sensitivity is associated with the heating range and birefringence of the fiber. When the outside temperature changes, the interference spectrum of the Lyot–Sagnac loop will be shifted upon Vernier amplification.

## 3. Results

**Figure 4:**
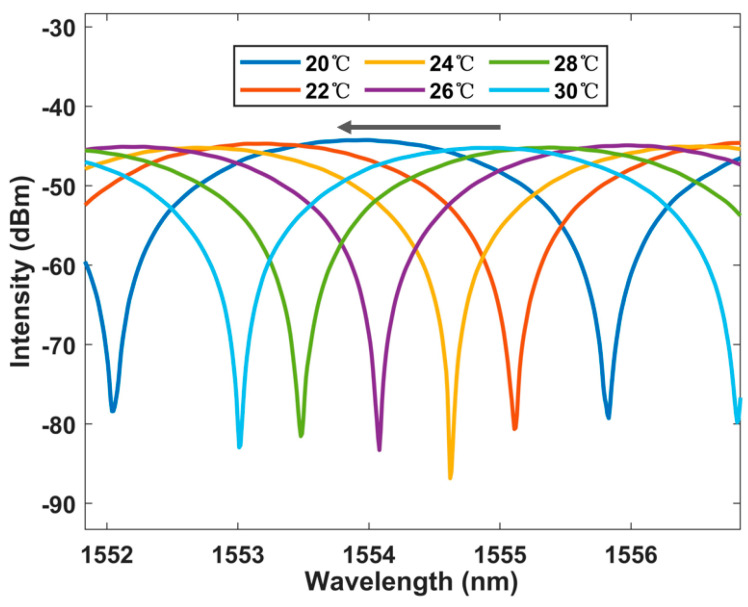
The output interference spectrum changes with temperature under BBS in the traditional Sagnac loop.

**Figure 5:**
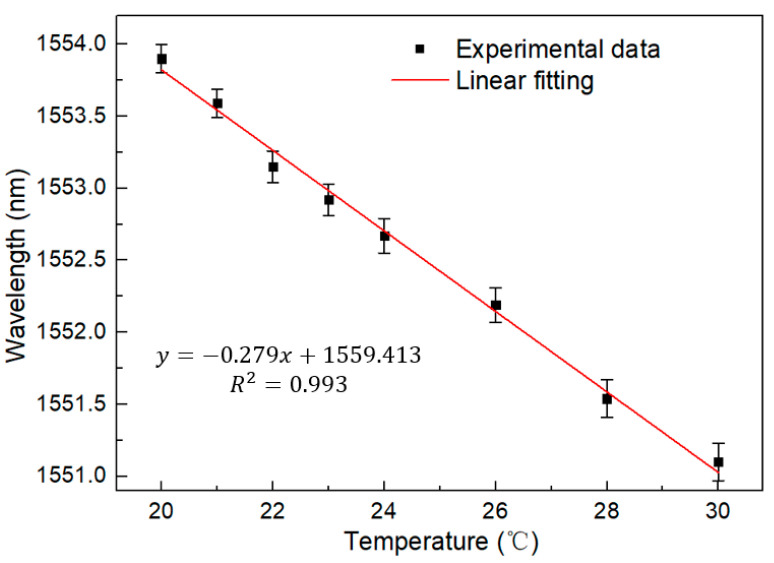
Linear regression equation of traditional Sagnac loop under temperature change in BBS at the temperature range from 20 °C to 30 °C.

The output spectrum of the BBS is shown in Figure 4. It can be seen that the interference spectrum generated in the traditional single Sagnac loop is very flat at the temperature range of 20–30 °C for a PMF length equal to 0.8 m. With the change in temperature, the wavelength will blue shift to the short wavelength direction. Sensitivity here represents the wavelength shift with temperature variation. The linear fitting curve is shown in Figure 5. Through analysis, the temperature response is −0.279 nm/°C. The fitting coefficient is as high as 0.993, which proves its good linearity.

**Figure 6:**
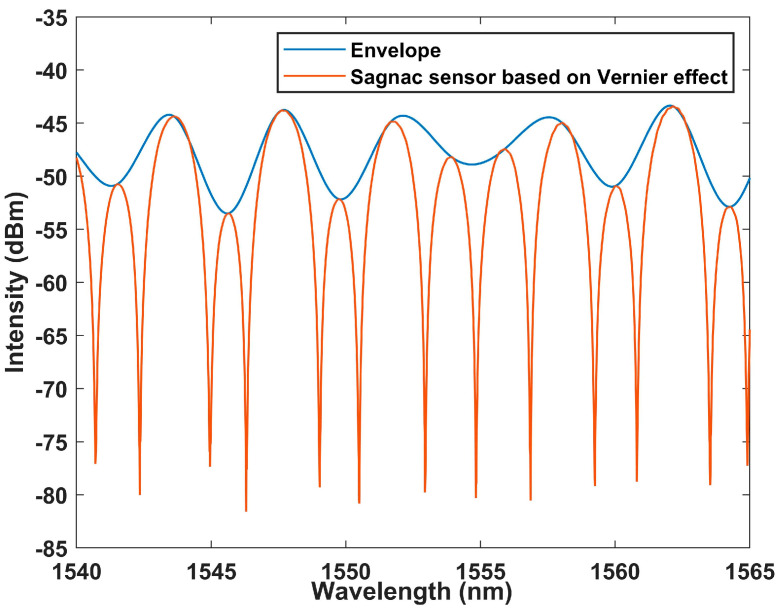
The correlation between the Lyot–Sagnac loop interference spectrum and its envelope.

Then, we tested the interference spectrum when the fast axis angle between two PMFs was 45°, and an extremely obvious Vernier envelope could be seen, as shown in Figure 6. This means that the overall envelope shift due to temperature changes may be amplified. The envelope generated by the spectral map of the Vernier effect is plotted using Matlab software (R2023a). In the beginning, we set ‘yEnv = zeros(13,000, 6)’. YEnv is a function used to retrieve the envelope in software. A value of 13,000 is our sampling point number; 13,000 points are sampled for each temperature group. The six represents six sets of data sampled at intervals of 2 °C from 20 °C to 30 °C. Then, we cycle through six samples to obtain envelope data at six temperatures, with 1500 sampling points, to ensure the accuracy of envelope drawing as much as possible.

**Figure 7:**
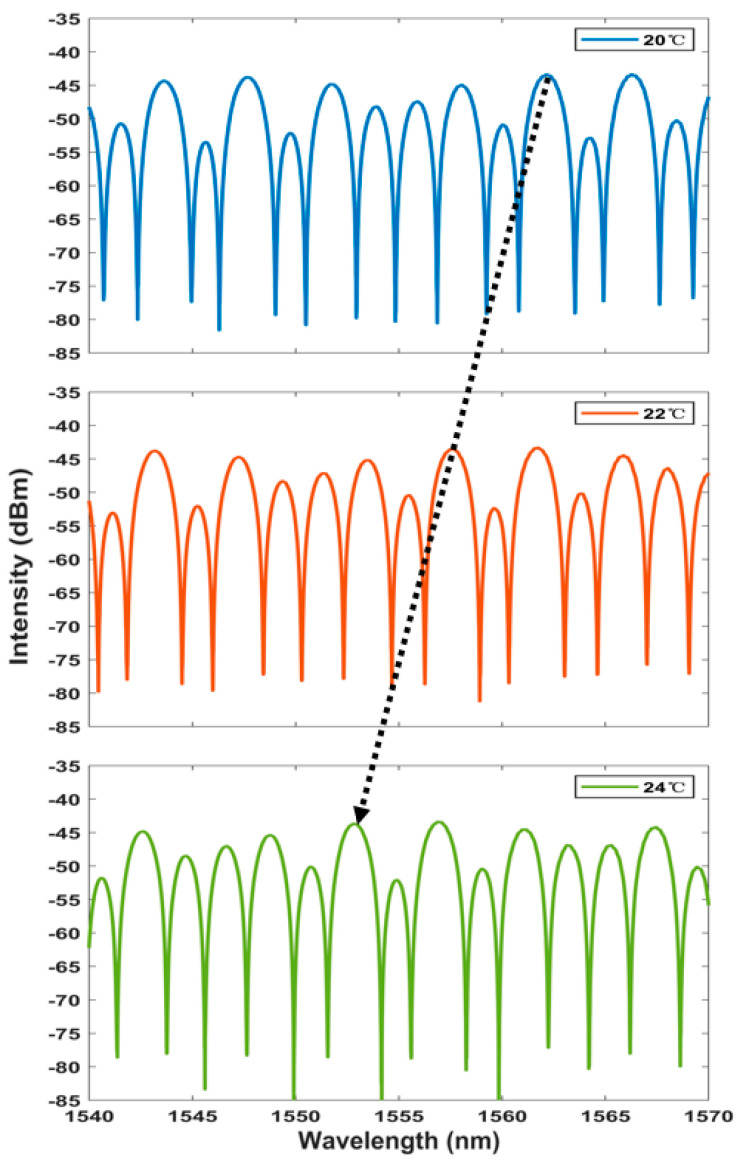
Broadband spectrum shift with temperature in the Lyot–Sagnac loop [blue: 20 °C, red: 22 °C, green: 24 °C].

**Figure 8:**
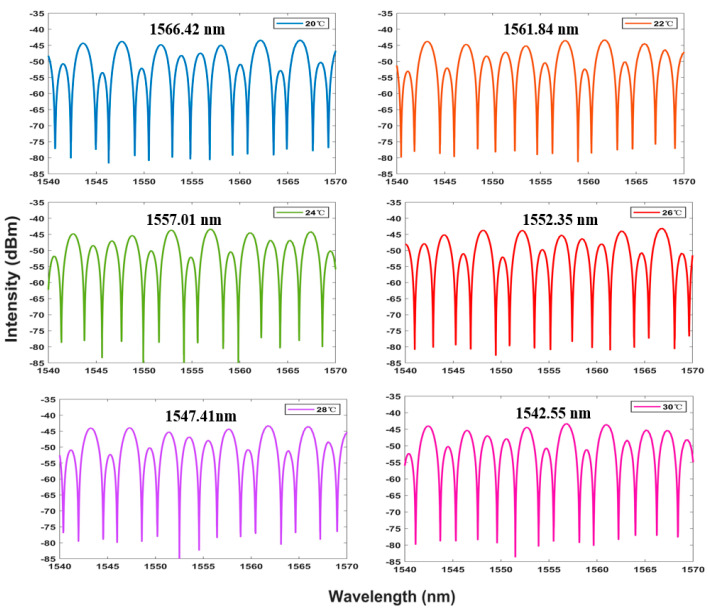
The output spectrum changes with temperature under BBS in the Lyot–Sagnac loop from 20 °C to 30° C.

**Figure 9:**
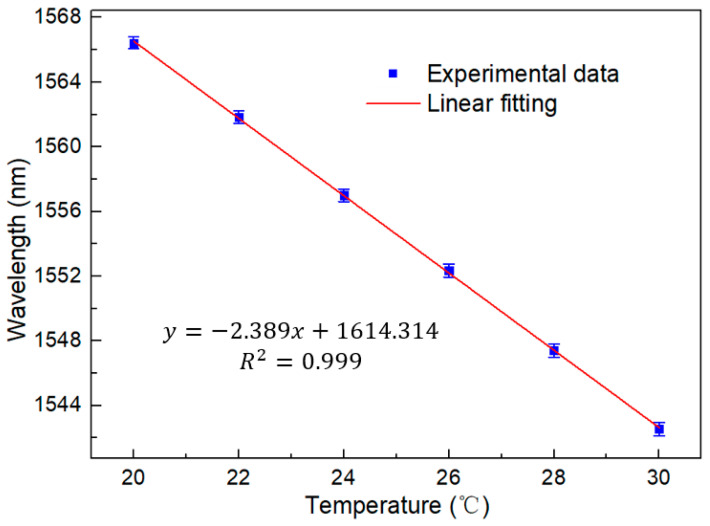
Linear regression equation of the Lyot–Sagnac loop under temperature change from 20 °C to 30 °C in BBS.

The spectrum generated using the Vernier envelope is shown in Figure 7. It can be seen that with the increase in temperature, the peak power of the spectrum will have a large shift. The curve of the Lyot–Sagnac loop spectrum changing with temperature under the detection limit of 20 °C to 30 °C is shown in Figure 8. The wavelength offset can also be read out through the figure. It can be found that with the increase in temperature, the wavelength shifts to the short wavelength, and the change is more significant than the traditional structure. In addition, the linear regression curve shown in Figure 9 shows that the sensitivity of the sensing system is as high as −2.389 nm/°C. The fitting coefficient is 0.999. This is 8.6 times more sensitive than the traditional single-ring structure. It shows good temperature response characteristics.

**Figure 10:**
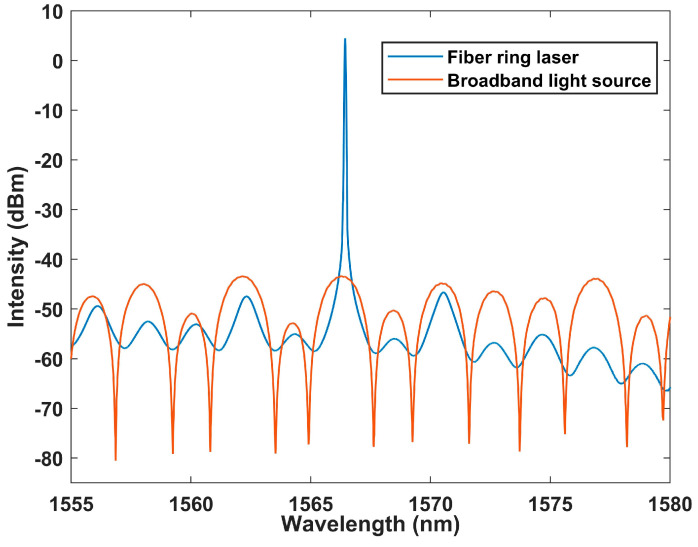
The output spectrum of laser and generated vernier envelope.

Then we further test the correspondence between the interference spectrum and the laser, as shown in Figure 10. It can be seen that the laser output is at the maximum intensity of the interference peak. In our preliminary experiment, we conducted temperature testing on the Lyot–Sagnac loop using a broadband light source, verifying its interference spectrum and its shift with temperature changes. This means that when the Lyot–Sagnac loop is placed in the laser cavity, the output wavelength of the laser can be modulated, as the output laser wavelength will be excited at the point where the interference spectrum is coherent. Hence, the interference spectrum of the Lyot–Sagnac loop works as a filter for the fiber ring laser system. This means the structure can be used as a good filter for temperature monitoring in the FRL system.

**Figure 11:**
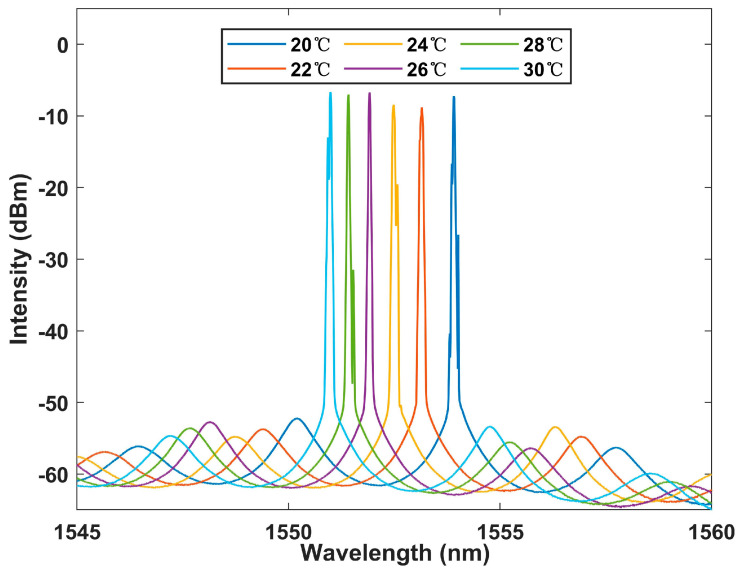
The output laser spectrum of traditional Sagnac loop structure at the temperature range of 20–30 °C in the FRL system.

**Figure 12:**
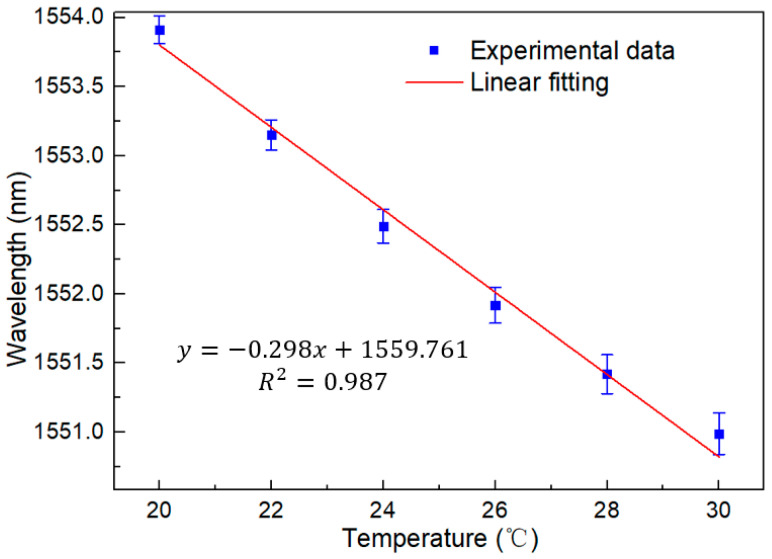
Linear regression equation of traditional Sagnac loop with a temperature change from 20 °C to 30 °C in the FRL system.

**Figure 13:**
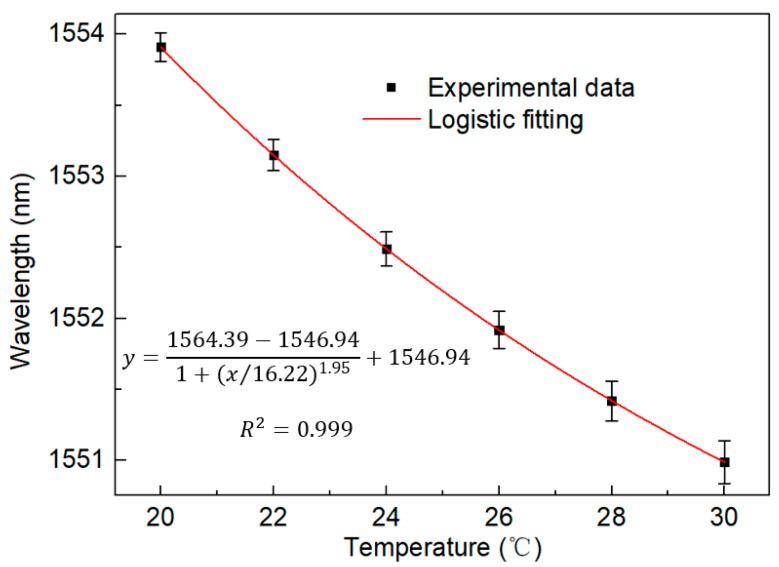
Nonlinear regression equation of traditional Sagnac loop 20 °C to 30 °C in the FRL system.

As can be seen from Figure 11, the laser output spectrum maintains good consistency with the temperature change in the BBS for the traditional Sagnac loop, with the wavelength moving towards the short wavelength direction, while the signal-to-noise ratio is up to 50 dB, and the line width is better than 0.2 nm. This is a unique advantage of laser sensing. In addition, Figure 12 shows the linear regression equation, and it can be seen that its sensitivity is −0.298 nm/°C, which is almost consistent with the sensitivity of the BBS system. In addition, the R squared value is 0.987. This proves that it has a good linearity in the FRL system. However, a quadratic fitting should be applied to the fitting to see how well it fits for further assessment of the analysis. The current linear fitting touched on the far end of the datapoint error bars for 20 °C, 24 °C, 26 °C, and 30 °C. This may be due to the incomplete matching between the peak of the laser output wavelength and the interference spectrum of the filter. The results are shown in Figure 13, and an excellent fitting curve can still be obtained for temperature monitoring.

**Figure 14:**
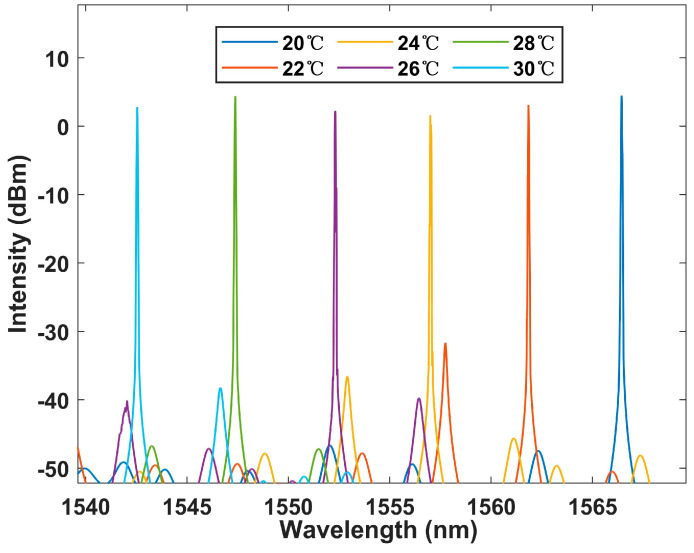
The Lyot–Sagnac loop structure output laser spectrum at the temperature range of 20–30 °C in the FRL system.

The Lyot–Sagnac loop was further placed into the laser cavity for experiments, and the results are shown in Figure 14. A more significant blue shift in the output spectrum can be seen, demonstrating the sensitization effect of the Lyot–Sagnac loop. In addition, the signal-to-noise ratio fits the SNR 50 dB performance, and the bandwidth is still better than 0.2 nm. This ensures its superiority over BBS systems.

**Figure 15:**
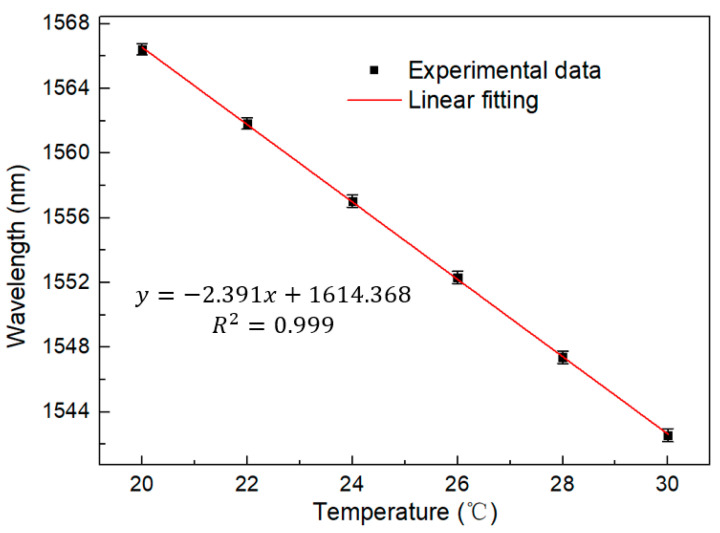
Linear regression equation of the Lyot–Sagnac loop with temperatures from 20 °C to 30 °C in the FRL system.

**Figure 16:**
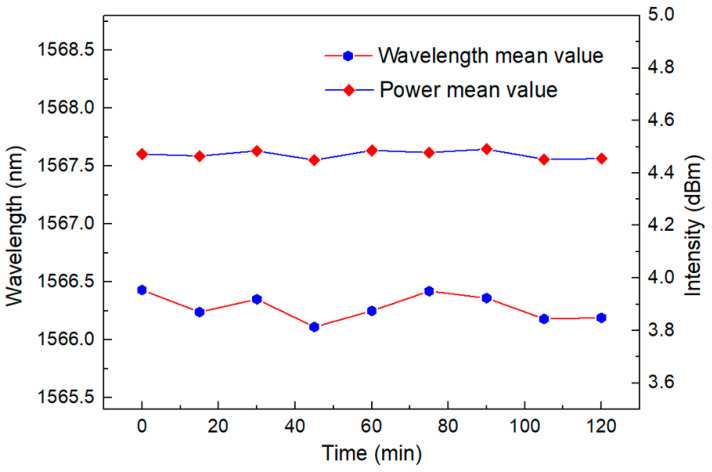
The stability of the Lyot–Sagnac loop as a temperature sensor in the FRL system is within a 2 h monitoring range. (When the temperature is 20 °C).

**Figure 17:**
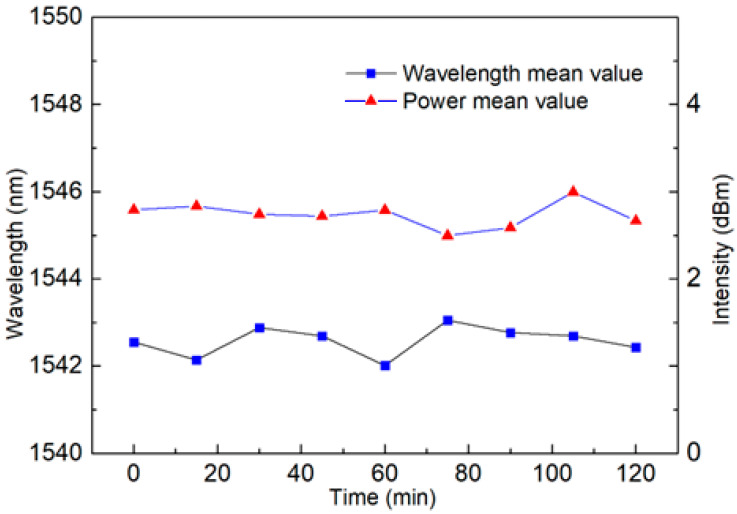
The stability of the Lyot–Sagnac loop as a temperature sensor in the FRL system is within a 2 h monitoring range. (When the temperature is 30 °C).

Figure 15 shows a linear regression equation with a detection sensitivity of −2.391 nm/°C. The temperature sensitivity is amplified eight times compared to a traditional single loop. At the same time, the R squared value is as high as 0.999. This proves its superior linearity, which is very important for a sensor. In addition, the stability of the sensor has also been verified through continuous monitoring at a temperature range of 20 °C and 30 °C for two hours, with a wavelength fluctuation range of less than 0.55 nm and an intensity fluctuation range of less than 0.1 dB, as shown in Figure 16 and Figure 17, in certain application scenarios, such as cell life detection, human body temperature monitoring, and temperature detection within limited timer operation, the stability of up to two hours can ensure the normal and accurate temperature monitoring.

Table 1 shows the sensitivity and maximum signal-to-noise ratio comparison results between the designed temperature sensor and various fiber optic temperature sensors in the past two years. It can be found that the sensor we designed has good sensitivity while maintaining a very high signal-to-noise ratio.

In addition, the resolution can be calculated through reference [45]; the temperature detection limit based on the laser is 0.0282 °C, while the detection limit based on the broadband light source is only 0.667 °C. The resolution of the laser cavity exceeds an order of magnitude. Although temperature sensitivity is particularly prominent in this study, it must be noted that its monitoring range is only 10 °C. Several factors limit its dynamic range. First, the gain range of the doped fiber is only a few tens of nanometers, beyond which the corresponding laser output cannot be obtained. Furthermore, there is more competition inside the laser cavity. Theoretically, after filtering through the Sagnac loop, laser output can be generated only at the frequency with the strongest interference peak. However, since the peak powers of the interference peak are very close to each other when beyond a certain range, the laser will jump and not move with the movement of the interference peak. Pulsation in a certain frequency range (about 20–40 nm). In fact, due to the widespread application of optical fiber in the field of communication, its price is very low, and typically thousands or even hundreds of Yuan can be purchased for thousands of kilometers of optical fiber. The length of the optical fiber used in this experiment is in the order of meters, so its cost is controlled within a hundred yuan. This is compared to other types of sensors, such as metamaterials and electronic materials. The cost is reduced by one to two orders of magnitude.

## 4. Conclusions

In this study, we proposed and demonstrated an ultra-high sensitivity temperature sensor in an FRL system. The system structure is simplified, and its sensitivity is significantly increased thanks to the Lyot–Sagnac loop’s ability to produce the Vernier amplification effect. The sensor is implemented upon rotating the fast axis of the two PMFs by 45°. The experimental results show that the sensitivity of the designed structure is up to −2.391 nm/°C in the temperature range of 20–30 °C, which is more than eight times higher than the sensitivity of the traditional Sagnac loop of −0.298 nm/°C. The stability of the system is shown by the wavelength fluctuation range being less than 0.39 nm and the intensity stability being better than 0.1 dB during the course of the two hour test period. Additionally, as a result of the FRL system’s properties, the output spectrum’s signal-to-noise ratio can reach 50 dB, and its bandwidth is smaller than 0.2 nm. The great sensitivity, straightforward design, and inexpensive cost of the suggested temperature sensor are benefits. It will have a vital role in detecting biological activity.

## Figures and Tables

**Table 1:** The sensitivity comparison with other optical fiber sensors.

Structures	Temperature Sensitivity (nm/°C)	Maximum Signal-to-Noise Ratio (dB)	Ref.
tapered-SHF MZI	0.427	40	[39]
SMF-FPI	0.014	10	[40]
FPI and MI	19.22	9	[41]
NCF-SPR	3.53	3	[42]
SPS MZI	15.8	35	[43]
MZI-SPR	1.425	3	[44]
This work	2.391	50	

## Data Availability

Not applicable.
